# Outcomes in Endovascular Therapy for Basilar Artery Occlusion: Intracranial Atherosclerotic Disease *vs*. Embolism

**DOI:** 10.14336/AD.2020.0704

**Published:** 2021-04-01

**Authors:** Longfei Wu, Gary B Rajah, Eric E Cosky, Xiling Wu, Chuanhui Li, Jian Chen, Wenbo Zhao, Di Wu, Yuchuan Ding, Xunming Ji

**Affiliations:** ^1^Department of Neurology and China-America Institute of Neuroscience, Xuanwu Hospital, Capital Medical University, Beijing, China.; ^2^Department of Neurosurgery, Jacobs School of Medicine and Biomedical Sciences, University at Buffalo, Buffalo, New York, USA.; ^3^Department of Neurosurgery, Gates Vascular Institute at Kaleida Health, Buffalo, New York, USA.; ^4^Department of Neurosurgery, Munson Healthcare, Traverse City, Michigan, USA.; ^5^Department of Neurosurgery, Wayne State University School of Medicine, Detroit, Michigan, USA.; ^6^Department of Traditional Chinese Medicine, Xuanwu Hospital, Capital Medical University, Beijing, China.; ^7^Department of Emergency, Xuanwu Hospital, Capital Medical University, Beijing, China.; ^8^Department of Neurosurgery, Xuanwu Hospital, Capital Medical University, Beijing, China.

**Keywords:** stroke, endovascular therapy, basilar artery occlusion, intracranial atherosclerotic disease, embolism

## Abstract

Acute ischemic stroke due to basilar artery occlusion (BAO) carries a very poor prognosis. Functional outcomes in BAO patients undergoing endovascular therapy (EVT) may differ according to the specific pathological mechanisms. We aimed to explore the impact of the underlying pathological mechanisms on prognosis at 90-days and long-term follow-up in BAO patients treated with EVT. We analyzed consecutive BAO patients undergoing EVT from December 2012 to December 2018 at a single center (Xuanwu Hospital). Patients were classified into either an intracranial atherosclerotic disease (ICAD) group or an embolic group according to the corresponding angiographic findings. The baseline characteristics and functional outcomes were compared between the two groups. Multivariable logistic regression analysis was performed. Among the 167 patients enrolled, 78 patients (46.7%) were in the ICAD group and 89 patients (53.3%) were assigned to the embolic group. Overall, 149 patients (89.2%) achieved successful reperfusion post-EVT. There were no significant differences in functional outcomes at 90-days and long-term follow-up between the two groups. Similarly, a Kaplan-Meier survival analysis showed similar long-term survival probabilities (P = 0.438). The pathological mechanism was not associated with functional independence (OR, 1.818; 95% CI, 0.694-4.761; P = 0.224), favorable outcome (OR, 1.476; 95% CI, 0.592-3.681; P = 0.403), or mortality (OR, 1.249; 95% CI, 0.483-3.226; P = 0.646). However, based on subgroup analysis, embolic BAO versus ICAD was significantly associated with better functional independence in those aged 60 years and younger (OR, 4.513; 95% CI, 1.138-17.902). In this study, no differences in either 90-days or long-term functional outcomes between ICAD-related BAO and embolic BAO patients undergoing EVT were observed. However, in BAO patients aged ≤ 60 years, the pathological mechanism of embolism was associated with better functional independence.

Acute ischemic stroke due to basilar artery occlusion (BAO) accounts for approximately 1% of all ischemic strokes and 5% of large vessel occlusions [1, 2]. BAO has a very poor prognosis, carrying a morbidity and mortality rate of over 80% [3-5]. Endovascular therapy (EVT) is an innovative revascularization method that has changed the treatment course for large vessel occlusions. With the implementation of modern endovascular devices and techniques, over 80% of EVT-treated patients achieve successful reperfusion [6, 7]. As the only multicenter randomized controlled trial investigating the efficacy and safety of EVT in BAO patients to date, Acute Basilar Artery Occlusion: Endovascular Interventions vs Standard Medical Treatment (BEST) trial was terminated prematurely due to excessive crossovers and a progressive drop in the average recruitment rate [8]. Although the intention-to-treat analysis in this trial failed to detect any differences in prognosis between patients receiving EVT versus those receiving standard medical therapy, secondary prespecified analyses in both per-protocol and as-treated populations supported the superiority of EVT. Additionally, a recent study indicated that EVT significantly improved the prognosis of BAO patients, as compared to standard medical treatment alone [9].

The most frequent causes of BAO are local thrombosis secondary to underlying intracranial atherosclerotic disease (ICAD) combined with plaque rupture and embolism from cardiac or large arteries [1, 10]. BAO patients with different pathological mechanisms may receive different therapeutic strategies during EVT, which could lead to improved functional outcomes. As such, it is crucial to accurately identify if the underlying pathological mechanisms of BAO contribute to distinct disease outcomes. Only a few studies have investigated the differences in prognosis post-EVT among BAO patients with different pathological mechanisms [11-13]. The small sample sizes and lack of long-term follow-up for functional outcomes are limitations of these previous studies. In the present study, we aimed to explore the impact of the underlying pathological mechanisms on prognosis at 90-days and long-term follow-up in BAO patients treated with EVT.

## MATERIALS AND METHODS

### Data availability statement

All supporting data contributing to this study can be provided on reasonable request.

### Registration and patient consent

This study was based on a prospective registry at Xuanwu Hospital, Capital Medical University in Beijing, China. As a comprehensive stroke center in Northern China, our institution receives and treats stroke patients from both primary and tertiary referral populations. Consecutive acute ischemic stroke patients undergoing reperfusion therapy (i.e. intravenous thrombolysis and EVT) have been registered in this prospectively collected database. This study was approved by the institutional review board at Xuanwu Hospital of Capital Medical University. Verbal and written informed consent were obtained at the time of admission by patients or their authorized legal representatives.

### Patient selection

For the present study, we enrolled all acute ischemic stroke patients admitted to our institution that underwent EVT from December 2012 to December 2018. The eligibility criteria were as follows: 1) acute ischemic stroke secondary to acute BAO or bilateral vertebral artery V4 segment occlusion leading to no blood flow to the basilar artery, as identified by computed tomographic angiography (CTA), magnetic resonance angiography (MRA), or digital subtraction angiography (DSA); 2) EVT was initiated (groin puncture) within 24 hours of stroke onset (or last known normal time); 3) patients needed a score of least 6 on the National Institutes of Health Stroke Scale (NIHSS) at baseline; and 4) patients were required to have a pre-morbid functional ability of 2 or less on the modified Rankin scale (mRS). The exclusion criteria included: 1) evidence of a large ischemic core as indicated by the posterior circulation Alberta Stroke Program Early Computed Tomography Score (pc-ASPECTS) of less than 6; 2) patients with a stroke in the distribution of the anterior circulation or a posterior cerebral artery occlusion; 3) occlusion resulting from other causes including vascular dissection, vasculitis, or Moyamoya disease; 4) patients with an indistinguishable pathological mechanism because reperfusion was never observed; or 5) patients who were lost to follow-up.

### Procedures

At our institution, acute ischemic stroke patients with large vessel occlusions receive EVT according to national guidelines [14]. The administration of intravenous alteplase prior to EVT was permitted for stroke patients who presented within 4.5 hours from the time of stroke onset. According to institutional protocols, only neurointerventionalists with extensive experience in neurovascular interventions were qualified to perform EVT for stroke patients. First and foremost, every patient underwent a four-vessel diagnostic DSA to identify the occluded artery and assess collateral blood flow in the affected vascular territory. The neurointerventionalist determined the device and procedural strategy to use based on the precise combination of the patient's condition and imaging results. The first-line approach of EVT consisted of mechanical thrombectomy with a stent retriever or contact aspiration devices. Intra-arterial thrombolysis, stenting, and balloon angioplasty were considered rescue therapies when the first-line procedure failed to achieve adequate reperfusion. After the procedure, patients treated with EVT were transferred to the neuro-intensive care and stroke units [14].

The underlying pathological mechanisms were classified into either ICAD or embolic by means of a thorough assessment of the source and maximum intensity projection reconstructed images from CTA and angiographic findings obtained during the procedures. In particular, ICAD was defined as significant stenosis at the occlusion site, which means that: 1) vessel stenosis > 70% or 2) stenosis > 50% with either impaired flow or perfusion on angiography or propensity to re-occlude despite EVT [15]. An embolism was defined as a lack of re-occlusion and significant stenosis after EVT with adequate perfusion. In this study, there were two blinded, experienced neurologists, who independently reviewed the neuroimaging results and determined the underlying pathological mechanisms of BAO among patients. The final determination was reached by consensus for cases with clinical discrepancies or where the mechanism was difficult to determine.

### Data collection

Data regarding patient demographics, comorbidities, stroke severity (NIHSS score), non-contrast CT findings (pc-ASPECTS), level of consciousness (GCS score), time intervals, occlusion sites, collateral status (American Society of Interventional and Therapeutic Neuroradiology/Society of Interventional Radiology [ASITN/SIR] scale), interventional procedure details, reperfusion condition (modified Thrombolysis in Cerebral Infarction [mTICI] perfusion score), and outcomes were all collected. The NIHSS score, with a full score being forty-two, indicates probable neurological deficits. A higher score on the NIHSS is consistent with more severe neurological disability [16]. The pc-ASPECTS ranges from 0-10 and is a semi-quantitative topographic assessment of early ischemic changes in the posterior circulation of the brain. A lower score indicates more extensive ischemia [17]. The GCS score ranges from 3-15 and assesses a patient’s level of consciousness. A lower score indicates a lower level of consciousness [18]. According to angiographic findings, we classified the vascular occlusion sites into intracranial vertebral artery (from the level of the vertebral artery V4 segment to just below the vertebrobasilar junction), proximal basilar artery (from the level of the vertebrobasilar junction to the anterior inferior cerebellar artery [AICA]), middle basilar artery (from the AICA to the superior cerebellar artery [SCA]), and distal basilar artery (distal to the SCA) [3]. The angiographic collateral scale known as ASITN/SIR assigns patients to six grades from 0 to 5 with lower grades corresponding to worse collateral blood flow [19]. The mTICI is an angiographic recanalization scale, ranging from grade 0 to 3. A grade of 2b or 3 indicates successful reperfusion with grade 3 signifying complete reperfusion [20-22].

### Outcomes assessment

The primary outcome was determined using the mRS score at 90 days after EVT. The mRS is an ordinal scale, ranging from 0 to 6. A score of 0 to 2 correlates with functional independence, 0 to 3 denotes a favorable outcome while 6 indicates mortality. We defined our secondary clinical outcomes as the rates of functional independence, favorable outcome, and mortality at 90-days follow-up along with long-term follow-up. Our secondary safety endpoints included procedure-related complications and serious adverse events. The procedural complications included vessel dissection, distal thrombus, perforation, vasospasm, and any hemorrhages. We identified serious adverse events, such as symptomatic intracranial hemorrhage, new ischemic stroke, progression of stroke, and pneumonia. An outcome assessment was performed by a blinded independent investigator through clinical visits or standardized telephone interviews with patients or their relatives.

### Statistical analyses

The baseline characteristics and outcomes were compared between the ICAD and embolic groups. Descriptive statistics are presented as the mean (standard deviation [SD]) for normally distributed continuous variables and the median (interquartile range [IQR]) for non-normally distributed continuous variables. The normality of distributions was assessed using the Kolmogorov-Smirnov test. Categorical variables are represented as percentages. In-between group comparisons were performed with either the Mann-Whitney U-test or student’s t-test for continuous variables or a chi-square test or Fisher's exact test for categorical variables, as statistically appropriate. In order to maintain the integrity of the data, we used single imputation to compensate for missing values that account for less than 5% of the total amount of variables. Next, to adjust for confounders, imbalanced baseline variables and known clinically relevant factors were entered in the multivariable logistic regression analysis model. We defined the pathological mechanism as the independent variable while functional outcome at 90 days was set as the dependent variable. There was a collection of covariates like age, atrial fibrillation, collateral status, NIHSS, occlusion sites, pc-ASPECTS, time from onset of stroke to reperfusion, and reperfusion status. Furthermore, a Kaplan-Meier survival analysis was performed using the log-rank test to compare the survival probabilities at long-term follow-up between the two groups. A two-sided P < 0.05 was considered as statistically significant. All statistical analyses were performed using IBM SPSS Statistics 26 (IBM Corp, Armonk, NY, USA).

**Table 1:** Baseline characteristics.

	Overall (n=167)	ICAD (n=78)	Embolism (n=89)	P value
Patient characteristics				
Age, y, mean (SD)	60.0 (11.8)	58.5 (9.5)	61.3 (13.3)	0.112
Male, n (%)	138 (82.6)	66 (84.6)	72 (80.9)	0.527
BMI, mean (SD)	26.0 (3.2)	26.0 (3.1)	26.0 (3.3)	0.897
NIHSS, median (IQR)	23 (15-33)	22 (15-34)	24 (15-33)	0.665
pc-ASPECTS, median (IQR)	8 (7-10)	8 (7-10)	8 (7-9)	0.593
GCS, median (IQR)	8 (5-12)	7 (5-12)	8 (5-11)	0.795
Intravenous alteplase, n (%)	34 (20.4)	14 (17.9)	20 (22.5)	0.469
Systolic BP, median (IQR)	150 (133-170)	146 (130-168)	150 (139-170)	0.323
Diastolic BP, median (IQR)	87 (76-95)	85 (75-94)	88 (79-96)	0.334
Comorbidities, n (%)				
Hypertension	131 (78.4)	59 (75.6)	72 (80.9)	0.410
Diabetes mellitus	48 (28.7)	27 (34.6)	21 (23.6)	0.116
Hyperlipidemia	41 (24.6)	15 (19.2)	26 (29.2)	0.135
Atrial fibrillation	29 (17.4)	3 (3.8)	26 (29.2)	<0.001
Smoking	77 (46.1)	38 (48.7)	39 (40.4)	0.526
Time intervals, min				
From stroke onset to groin puncture, median (IQR)	557 (365-783)	578 (334-782)	540 (381-798)	0.890
From groin puncture to reperfusion, median (IQR)	60 (46-78)	72 (55-85)	55 (40-74)	<0.001
From stroke onset to reperfusion, median (IQR)	621 (424-849)	654 (402-851)	579 (434-844)	0.655
General anesthesia, n (%)	103 (61.7)	45 (57.7)	58 (65.2)	0.321
Occlusion sites, n (%)				
Intracranial VA	25 (15.0)	15 (19.2)	10 (11.2)	0.149
Proximal BA	51 (30.5)	46 (59.0)	5 (5.6)	<0.001
Middle BA	22 (13.2)	9 (11.5)	13 (14.6)	0.559
Distal BA	69 (41.3)	8 (10.3)	61 (68.5)	<0.001
Collateral status, n (%)				
ASITN/SIR grade 0-2	122 (73.1)	47 (60.3)	75 (84.3)	<0.001
ASITN/SIR grade 3-4	45 (26.9)	31 (39.7)	14 (15.7)	
Number of passes, median (IQR)	1 (1-2)	2 (1-2)	1 (1-2)	0.192
Interventional procedures, n (%)				
Stent retriever	138 (82.6)	67 (85.9)	71 (79.8)	0.297
Aspiration	47 (28.1)	20 (25.6)	27 (30.3)	0.501
Intra-arterial thrombolysis	25 (15.0)	12 (15.4)	13 (14.6)	0.888
Stenting	59 (35.3)	37 (47.4)	22 (24.7)	0.002
Balloon angioplasty	31 (18.6)	22 (28.2)	9 (10.1)	0.003
Reperfusion, n (%)				
mTICI ≥ 2b	149 (89.2)	66 (84.6)	83 (93.3)	0.072
mTICI 3	78 (46.7)	31 (39.7)	47 (52.8)	0.091

Abbreviations: ICAD, intracranial atherosclerotic disease; SD, standard deviation; BMI, body mass index; NIHSS, National Institutes of Health Stroke Scale; IQR, interquartile range; pc-ASPECTS, posterior circulation Alberta Stroke Program Early Computed Tomography Score; GCS, Glasgow Coma Scale; BP, blood pressure; VA, vertebral artery; BA, basilar artery; ASITN/SIR, American Society of Interventional and Therapeutic Neuroradiology/Society of Interventional Radiology; mTICI, modified thrombolysis in cerebral infarction.

## RESULTS

A total of 546 acute ischemic stroke patients were admitted to our institution and underwent EVT from December 2012 to December 2018. There were 379 patients that were excluded after applying the eligibility and exclusion criteria. Therefore, the cohort consisted of 167 patients, which included 78 patients (46.7%) in the ICAD group and 89 patients (53.3%) in the embolism group.

### Baseline characteristics

The baseline characteristics of the cohort are summarized in Table 1. Of the 167 enrolled patients, the mean age was 60 years (SD, 11.8) and 138 patients (82.6%) were male. The median GCS, NIHSS, and pc-ASPECTS scores were 8 (IQR, 5-12), 23 (IQR, 15-33), and 8 (IQR, 7-10), respectively. A total of thirty-four patients (20.4%) were administered intravenous alteplase prior to EVT.

Among the comorbidities, twenty-nine patients (17.4%) had atrial fibrillation. Atrial fibrillation was more prevalent in patients of the embolism group, as compared to the ICAD group (3.8% vs 29.2%; P < 0.001). In terms of time intervals, the median time from stroke onset to groin puncture, groin puncture to reperfusion, and stroke onset to reperfusion were 557 min (IQR, 365-783), 60 min (IQR, 46-78), and 621 min (IQR, 424-849), respectively. In comparison to the embolism group, patients in the ICAD group encountered a longer time from groin puncture to reperfusion (72 min [IQR, 55-85] vs 55 min [IQR, 40-74]; P < 0.001). An occlusion of the proximal basilar artery was more commonly observed among patients in the ICAD group (59% vs 5.6%; P < 0.001), whereas distal basilar artery occlusion was more frequently found within patients of the embolism group (10.3% vs 68.5%; P < 0.001). The assignment of ASITN/SIR grades 3-4 to patients in the ICAD group was significantly higher than those in embolism group (39.7% vs 15.7%; P < 0.001), thus suggesting that patients with BAO secondary to ICAD had better collateral vessel status.

**Table 2:** Primary and secondary outcomes.

	Overall (n=167)	ICAD (n=78)	Embolism (n=89)	P value
Primary outcome, median (IQR)				
mRS at 90 days	4 (2-6)	5 (2-6)	4 (2-6)	0.802
Secondary outcomes, clinical, n (%)				
90-day follow-up				
Functional independence	51 (30.5)	21 (26.9)	30 (33.7)	0.342
Favorable outcome	70 (41.9)	31 (39.7)	39 (43.8)	0.594
Mortality	53 (31.7)	23 (29.5)	30 (33.7)	0.559
Long-term follow-up				
Functional independence	65 (38.9)	29 (37.2)	36 (40.4)	0.665
Favorable outcome	73 (43.7)	33 (42.3)	40 (44.9)	0.732
Mortality	65 (38.9)	28 (35.9)	37 (41.6)	0.453
Secondary outcomes, safety, n (%)				
Procedure-related complications	36 (21.6)	17 (21.8)	19 (21.3)	0.944
Serious adverse events				
Symptomatic intracranial hemorrhage	10 (6.0)	5 (6.4)	5 (5.6)	0.830
New ischemic stroke	6 (3.6)	3 (3.8)	3 (3.4)	1
Progression of stroke	11 (6.6)	7 (9.0)	4 (4.5)	0.244
Pneumonia	46 (27.5)	24 (30.8)	22 (24.7)	0.383

During the procedure, 138 patients (82.6%) underwent EVT using a stent retriever as the first-line approach. The rate of stent retriever use did not differ between the two groups. However, as rescue therapy, stenting (47.4% vs 24.7%; P = 0.002) and balloon angioplasty (28.2% vs 10.1%; P = 0.003) were more frequently used in patients from the ICAD group than the embolism group. In terms of reperfusion, a trend toward a higher rate of successful reperfusion was observed for patients in the embolism group (84.6% vs 93.3%; P = 0.072).

### Outcomes

**Figure 1.**
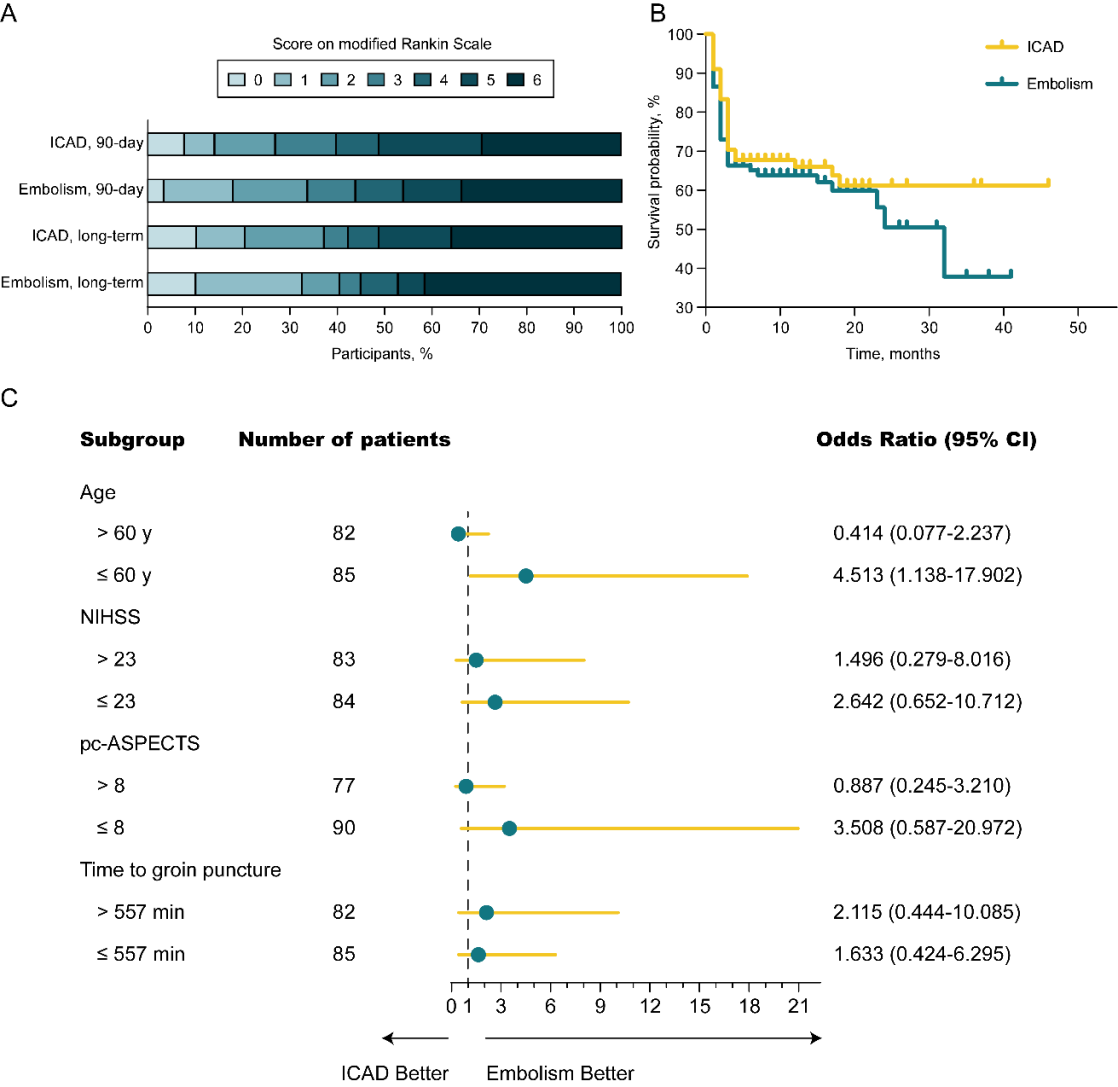
Functional outcomes, survival probability, and subgroups analysis. (A) No significant difference was observed in the distribution of modified Rankin Scale between the ICAD and embolism groups at either 90-days (P = 0.802) or long-term follow-up (P = 0.798). (B) There was no significant survival difference between the ICAD and embolism groups at long-term follow-up (log-rank, P = 0.438). (C) A forest plot shows that, in patients aged 60 years and younger, the difference in the clinical outcomes favored the embolism group (multivariable logistic regression analysis). However, there were no significant differences between the two groups among other subgroups. The cutoff values and categories of the subgroups were based on the median values. Abbreviations: ICAD, intracranial atherosclerotic disease; CI, Confidence Interval; NIHSS, National Institutes of Health Stroke Scale; pc-ASPECTS, posterior circulation Alberta Stroke Program Early Computed Tomography Score.

Outcomes in the study cohort are shown in Figure 1A and Table 2. Of the 167 patients, the median mRS score at 90 days post-EVT was 4 (IQR, 2-6). The ICAD and embolism groups had comparable 90-day mRS scores (5 [IQR, 2-6] vs 4 [2-6]; P = 0.802). No significant differences in the rates of functional independence, favorable outcome, or mortality at 90-days and long-term follow-up (mean observation period = 12 months) were observed between the two groups. A Kaplan-Meier survival analysis showed similar long-term survival probabilities between the two groups (log-rank; P = 0.438; Fig. 1B). Overall, ten patients (6%) had symptomatic intracranial hemorrhage. There were no significant differences between the two groups in terms of procedure-related complications and serious adverse events.

### Multivariable regression analysis

In the study cohort, after adjustment for related confounders, the underlying pathological mechanism was not associated with functional independence (OR, 1.818; 95% CI, 0.694-4.761; P = 0.224), favorable outcome (OR, 1.476; 95% CI, 0.592-3.681; P = 0.403), or mortality (OR, 1.249; 95% CI, 0.483-3.226; P = 0.646) in BAO patients at 90 days post-EVT. Age (OR, 0.956; 95% CI, 0.923-0.991; P = 0.014), NIHSS (OR, 0.932; 95% CI, 0.897-0.968; P < 0.001), pc-ASPECTS (OR, 1.535; 95% CI, 1.121-2.101; P = 0.008), and time from onset of stroke to reperfusion (OR, 0.998; 95% CI, 0.997-1.000; P = 0.016) were independently associated with functional independence. Next, NIHSS (OR, 0.924; 95% CI, 0.891-0.959; P < 0.001), pc-ASPECTS (OR, 1.570; 95% CI, 1.178-2.094; P = 0.002), and reperfusion condition (OR, 6.492; 95% CI, 1.186-35.543; P = 0.031) were independently associated with favorable outcome. Furthermore, the pc-ASPECTS (OR, 0.720; 95% CI, 0.549-0.944; P = 0.018), occlusion sites (OR, 0.619; 95% CI, 0.415-0.923; P = 0.019), NIHSS (OR, 1.057; 95% CI, 1.018-1.099; P = 0.004), and collateral status (OR, 0.241; 95% CI, 0.086-0.677; P = 0.007) were independently associated with mortality at 90-days follow-up. The results of the multivariable regression analysis are provided in Table 3.

**Table 3:** Predictors of functional outcomes at 90-day follow-up (multivariable analysis).

	Functional independence			Favorable outcome			Mortality		

	OR	95% CI	P value	OR	95% CI	P value	OR	95% CI	P value
Age	0.956	0.923-0.991	0.014	0.977	0.944-1.011	0.177	1.021	0.987-1.057	0.231
NIHSS	0.932	0.897-0.968	<0.001	0.924	0.891-0.959	<0.001	1.057	1.018-1.099	0.004
pc-ASPECTS	1.535	1.121-2.101	0.008	1.570	1.178-2.094	0.002	0.720	0.549-0.944	0.018
Atrial fibrillation	1.413	0.461-4.326	0.545	0.945	0.323-2.763	0.918	1.852	0.638-5.379	0.257
Time from stroke onset to reperfusion	0.998	0.997-1.000	0.016	0.999	0.997-1.000	0.088	1.001	0.999-1.002	0.229
Occlusion sites	0.886	0.599-1.311	0.546	0.986	0.675-1.439	0.941	0.619	0.415-0.923	0.019
Collateral status	1.482	0.598-3.673	0.386	2.162	0.886-5.278	0.090	0.241	0.086-0.677	0.007
Reperfusion condition	2.749	0.495-15.268	0.248	6.492	1.186-35.543	0.031	0.733	0.234-2.296	0.594
Pathological mechanism	1.818	0.694-4.761	0.224	1.476	0.592-3.681	0.403	1.249	0.483-3.226	0.646

Abbreviations: OR, Odds Ratio; CI, Confidence Interval; NIHSS, National Institutes of Health Stroke Scale; pc-ASPECTS, posterior circulation Alberta Stroke Program Early Computed Tomography Score.

### Subgroup analysis

Subsequently, a subgroup analysis was performed to investigate the relationship between the pathological mechanism and 90-days functional outcome. The cutoff values and subgroup categories were based on the median values. Among patients aged 60 years and younger, the pathological mechanism of embolism was significantly associated with functional independence (OR, 4.513; 95% CI, 1.138-17.902; P = 0.032). The baseline characteristics and outcomes among these patients are displayed in Tables 4 and 5, respectively. However, there was no significant correlation between pathological mechanism and functional independence in the other subgroups. The details of the subgroup analysis are presented in Figure 1C.

**Table 4:** Baseline characteristics among patients aged 60 years and younger.

	Overall (n=85)	ICAD (n=45)	Embolism (n=40)	P value
Patient characteristics				
Age, y, mean (SD)	51.2 (7.9)	52.4 (7.3)	49.7 (8.4)	0.115
Male, n (%)	77 (90.6)	40 (88.9)	37 (92.5)	0.569
BMI, mean (SD)	25.9 (3.5)	26.1 (3.2)	25.6 (3.9)	0.519
NIHSS, median (IQR)	24 (16-35)	23 (16-34)	25 (15-37)	0.454
pc-ASPECTS, median (IQR)	8 (8-10)	8 (8-10)	8 (7-9)	0.831
GCS, median (IQR)	6 (4-12)	6 (4-13)	7 (4-12)	0.821
Intravenous alteplase, n (%)	18 (21.2)	11 (24.4)	7 (17.5)	0.434
Systolic BP, median (IQR)	150 (130-170)	147 (130-169)	154 (140-170)	0.346
Diastolic BP, median (IQR)	88 (81-100)	85 (81-97)	90 (81-100)	0.417
Comorbidities, n (%)				
Hypertension	68 (80.0)	35 (77.8)	33 (82.5)	0.587
Diabetes mellitus	23 (27.1)	13 (28.9)	10 (25.0)	0.687
Hyperlipidemia	15 (17.6)	5 (11.1)	10 (25.0)	0.094
Atrial fibrillation	7 (8.2)	1 (2.2)	6 (15.0)	0.048
Smoking	58 (68.2)	32 (71.1)	26 (65.0)	0.546
Time intervals, min				
From stroke onset to groin puncture, median (IQR)	510 (330-734)	522 (331-731)	449 (290-750)	0.619
From groin puncture to reperfusion, median (IQR)	60 (47-79)	72 (49-83)	59 (39-76)	0.041
From stroke onset to reperfusion, median (IQR)	580 (398-791)	596 (401-791)	522 (361-793)	0.526
General anesthesia, n (%)	51 (60.0)	26 (57.8)	25 (62.5)	0.657
Occlusion sites, n (%)				
Intracranial VA	11 (12.9)	8 (17.8)	3 (7.5)	0.159
Proximal BA	28 (32.9)	26 (57.8)	2 (5.0)	<0.001
Middle BA	12 (14.1)	4 (8.9)	8 (20.0)	0.142
Distal BA	34 (40.0)	7 (15.6)	27 (67.5)	<0.001
Collateral status, n (%)				
ASITN/SIR grade 0-2	61 (71.8)	28 (62.2)	33 (82.5)	0.038
ASITN/SIR grade 3-4	24 (28.2)	17 (37.8)	7 (17.5)	
Interventional procedures, n (%)				
Stent retriever	75 (88.2)	41 (91.1)	34 (85.0)	0.383
Aspiration	25 (29.4)	12 (26.7)	13 (32.5)	0.556
Intra-arterial thrombolysis	8 (9.4)	6 (13.3)	2 (5.0)	0.272
Stenting	33 (38.8)	22 (48.9)	11 (27.5)	0.043
Balloon angioplasty	16 (18.8)	12 (26.7)	4 (10.0)	0.050
Reperfusion, n (%)				
mTICI ≥ 2b	77 (90.6)	39 (86.7)	38 (95)	0.272
mTICI 3	37 (43.5)	18 (40.0)	19 (47.5)	0.486

Abbreviations: ICAD, intracranial atherosclerotic disease; SD, standard deviation; BMI, body mass index; NIHSS, National Institutes of Health Stroke Scale; IQR, interquartile range; pc-ASPECTS, posterior circulation Alberta Stroke Program Early Computed Tomography Score; GCS, Glasgow Coma Scale; BP, blood pressure; VA, vertebral artery; BA, basilar artery; ASITN/SIR, American Society of Interventional and Therapeutic Neuroradiology/Society of Interventional Radiology; mTICI, modified thrombolysis in cerebral infarction.

## DISCUSSION

In the current study, we investigated the impact of the underlying pathological mechanism on the prognosis of patients with BAO that were treated with EVT. Despite baseline characteristic differences that existed between patients of the two groups, we did not identify any existed, overall significant differences in 90-days or long-term functional outcomes between them. Remarkably, in patients 60 years old and younger, embolism was independently associated with better functional independence. Given the low incidence of BAO, to our knowledge, this study is one of the largest to date that explores the different pathological mechanisms in patients with BAO undergoing EVT. The present study included long-term functional outcomes, which is a beneficial supplement to many of the previous studies.

**Table 5:** Primary and secondary outcomes among patients aged 60 years and younger.

	Overall (n=85)	ICAD (n=45)	Embolism (n=40)	P value
Primary outcome, median (IQR)				
mRS at 90 days	4 (2-6)	4 (2-5)	3 (1-6)	0.382
Secondary outcomes, clinical, n (%)				
90-day follow-up				
Functional independence	30 (35.3)	12 (26.7)	18 (45.0)	0.077
Favorable outcome	38 (44.7)	17 (37.8)	21 (52.5)	0.173
Mortality	22 (25.9)	10 (22.2)	12 (30.0)	0.414
Long-term follow-up				
Functional independence	38 (44.7)	17 (37.8)	21 (52.5)	0.173
Favorable outcome	41 (48.2)	20 (44.4)	21 (52.5)	0.458
Mortality	27 (31.8)	14 (31.1)	13 (32.5)	0.891
Secondary outcomes, safety, n (%)				
Procedure-related complications	22 (25.9)	12 (26.7)	10 (25.0)	0.861
Serious adverse events				
Symptomatic intracranial hemorrhage	6 (7.1)	2 (4.4)	4 (10.0)	0.414
New ischemic stroke	5 (5.9)	2 (4.4)	3 (7.5)	0.663
Progression of stroke	7 (8.2)	4 (8.9)	3 (7.5)	1
Pneumonia	25 (29.4)	13 (28.9)	12 (30.0)	0.911

Abbreviations: ICAD, intracranial atherosclerotic disease; IQR, interquartile range; mRS, modified Rankin Scale.

In particular, prior studies have suggested that the prognosis of BAO patients treated with EVT differed according to the underlying pathological mechanism. A literature review revealed that patients in the embolism group seem to have a more favorable outcome versus those in the ICAD group [12, 13]. This finding is inconsistent with our findings. The reason for the discrepancy between the groups may be due to differences in the status of collateral vessels. In our study, although a shorter intraoperative time during EVT along with a relatively higher rate of successful reperfusion appear to confer a better prognosis for patients in the embolism group, the adverse effects of the poorer collateral status may offset the benefits of shorter intraoperative duration and improved reperfusion. This likely results in no difference at 90-days or long-term outcomes between the groups. The posterior circulation consists predominantly of collateral vessels and a plethora of perforating arteries emanating from the basilar artery [23]. When BAO occurs, in the absence of adequate collateral blood flow, the brainstem, which is supplied by the perforating arteries of the basilar artery, will undergo time-sensitive and irreversible destruction in the case of continuous occlusion. The impact of collateral vessel status on the prognosis of patients with BAO has been observed in an earlier cohort study [24]. Similarly, our study demonstrated that poor collateral vascular status was significantly associated with mortality (Table 3). We identified a significant association between embolism and functional independence among patients with BAO that were 60 years old and younger. Perhaps, this is because younger stroke patients have a relatively better prognosis post-EVT [25], which further increases the difference in prognosis among patients with different pathological mechanisms of stroke. A retrospective study also revealed that younger patients with embolic BAO were more likely to benefit from thrombolysis [26]. Future investigations exploring this issue will need to extrapolate this finding.

In the era of pre-reperfusion therapy, patients with an embolic BAO were considered to have the worst prognosis, as they had no time for collaterals to develop [27, 28]. The implementation of modern endovascular devices and techniques has made it possible to efficiently recanalize the occluded basilar artery, especially in patients with emboli. From this perspective, the emergence of EVT benefits embolic BAO patients more than ICAD BAO patients.

According to our findings, the proximal basilar artery was more commonly occluded as a consequence of ICAD, while distal occlusion was more frequently caused by emboli. These findings are in accordance with previously reported results [12, 27, 29]. Anatomically, the pons receives blood from the proximal and middle segments of the basilar artery [1], thus extensive ischemia of the pons develops when proximal BAO occurs, which is theoretically worse than distal BAO. However, we did not find support for this in the present study. Likely, the distal collateral status among ICAD patients who were characterized by the proximal BAO explains this finding.

A longer intraoperative period in patients with BAO and ICAD was attributed to the use of second-line balloon angioplasty and stent placement after EVT failure. Navigating a stent retriever or an aspiration catheter through an artery with atherosclerotic stenosis is always challenging, not to mention the irritation or damage of vascular endothelium, and subsequent possible plaque rupture caused by the passage of endovascular devices through the stenosis would greatly increase the possibility of early re-occlusion [30]. In such cases, repeated mechanical thrombectomy and or additional rescue therapy (i.e. stent placement or balloon angioplasty) are critical to maintaining the reperfusion status, which will undoubtedly increase the intraoperative time during the procedure. Whether angioplasty and stenting are beneficial for BAO secondary to ICAD remains unknown. The negative results of the Stenting and Aggressive Medical Management for Preventing Recurrent Stroke in Intracranial Stenosis (SAMMPRIS) trial cast doubt on the safety of angioplasty and stenting in patients with atherosclerotic intracranial arterial stenosis [31, 32]. A recent research investigation, the Wingspan Stent System Post Market Surveillance (WEAVE) trial, found only a 2.6% risk of periprocedural stroke, hemorrhage, or death with 97.4% event freedom at 72 hours for patients with symptomatic ICAD who underwent stenting [33]. These results are promising and give prospect to future studies. Given the poor prognosis of patients with BAO, there needs to be more aggressive interventions to maintain their reperfusion status. A case series reported a recanalization rate of 100% and a 90-day functional independence rate of approximately 50% in patients with BAO and ICAD who received mechanical thrombectomy together with angioplasty and stenting in the same surgery [34]. In the future, there needs to be scientific studies that analyze and evaluate this issue.

Our investigation has several potential limitations related to its non-randomized nature. Despite using multivariable regression analysis to adjust for confounders and minimize biases, a potential bias might exist. Our study design at a single medical center may limit the generalizability of our findings, as stroke management varies among institutions internationally. The angiographic definition that we used to differentiate the specific pathological mechanism for each patient with BAO in our study may differ from other classification methods elsewhere. Therefore, clinicians should interpret our findings cautiously.

In conclusion, among stroke patients with BAO undergoing EVT, significant differences in 90-days and long-term functional outcomes between ICAD-related BAO and embolic BAO were not observed. However, in BAO patients aged 60 years old and younger, the pathological mechanism of embolism was independently associated with functional independence. Lastly, differentiating between these two pathologies has important implications for endovascular management, which makes positive clinical outcomes possible for both groups.
